# Supplementary material for: 10-Month-Old Infants Are Sensitive to the Time Course of Perceived Actions: Eye-Tracking and EEG Evidence
Source: Front Psychol. 2017 Jul 14;8:1170. doi: 10.3389/fpsyg.2017.01170 (PMC5509954; doi:10.3389/fpsyg.2017.01170)
Supplement: Supplementary file 1 [file Image_1.PDF]

Supplementary Figure 1

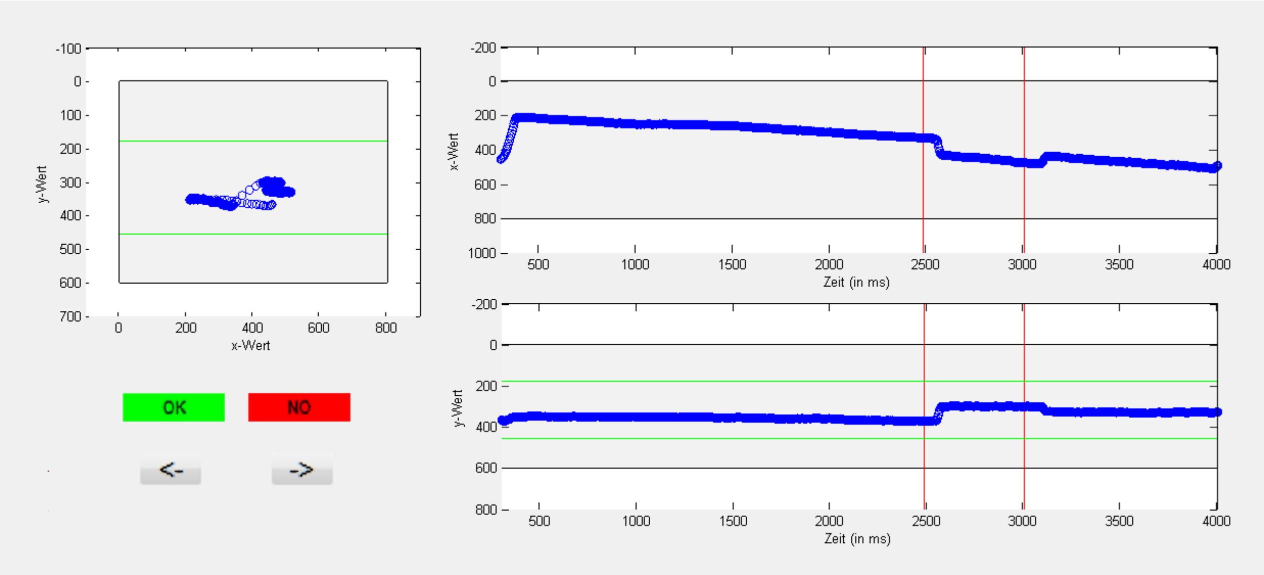

Supplementary Figure 1. Graphical User Interface used for visual inspection of eye-tracking data showing an exemplary trial. Upper left – spatial distribution of raw gaze points; upper right – raw x-values across time, lower right – y-values across time. Green horizontal lines indicate the approximate size and position of target, red vertical lines indicate on- and offset of occlusion, and the gray area indicates monitor dimensions (800 x 600 pixel).
