# Supplementary material for: 10-Month-Old Infants Are Sensitive to the Time Course of Perceived Actions: Eye-Tracking and EEG Evidence
Source: Front Psychol. 2017 Jul 14;8:1170. doi: 10.3389/fpsyg.2017.01170 (PMC5509954; doi:10.3389/fpsyg.2017.01170)
Supplement: Supplementary file 2 [file Image_2.PDF]

Supplementary Figure 2

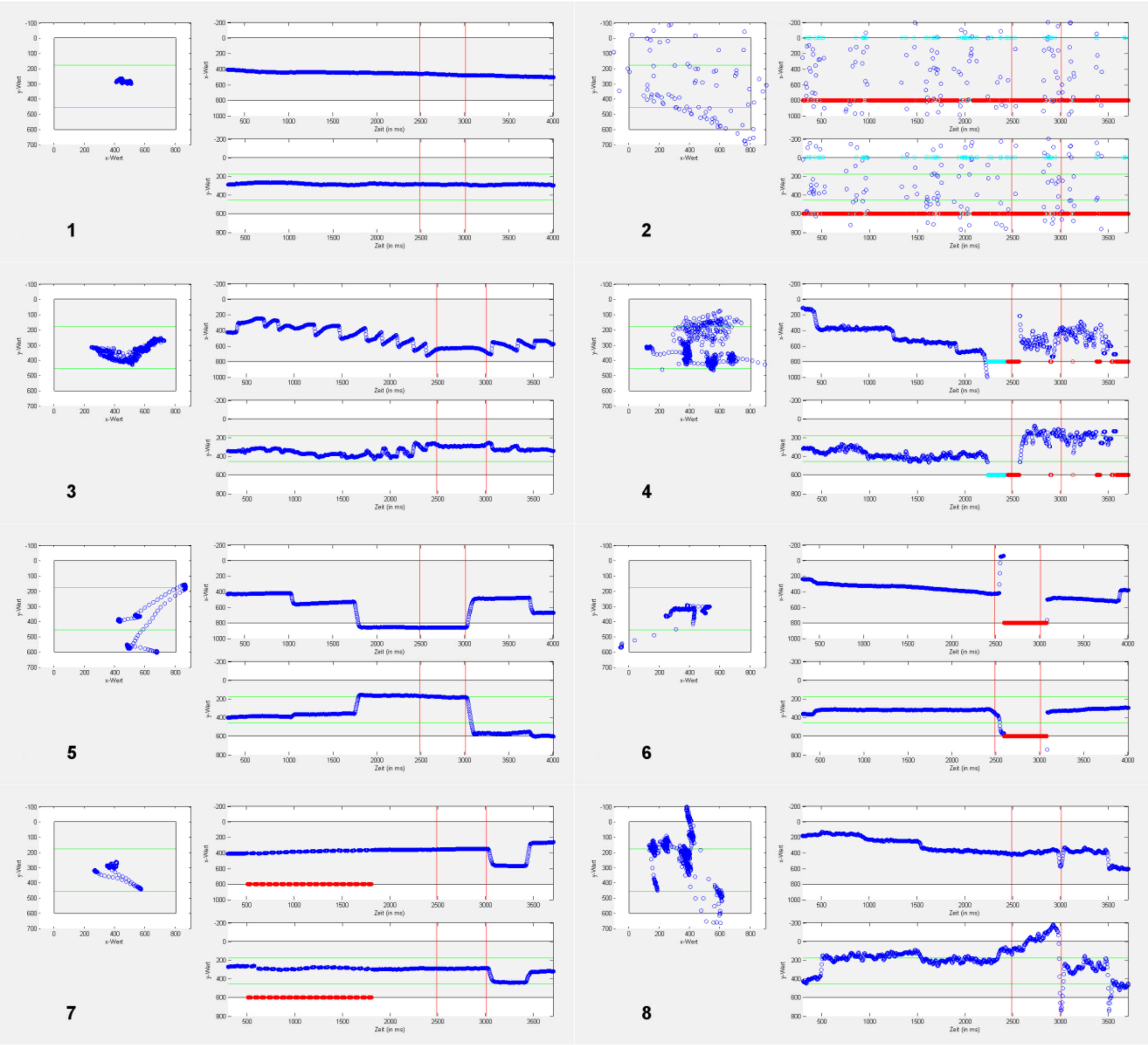

Supplementary Figure 2. Sample trials of raw gaze points illustrating low data quality discarded from analysis. Each display is shown in analogy to *Supplementary Figure 1*. Gaze points more than 200 pixel outside the monitor are displayed in cyan, missing values are in red. Criteria for exclusion (see also *2.3.1.2* of main text): *(a)* persistent or repeated missing, outlying, and/or close-to-border gaze points around occlusion phase (2, 4, 5, 7, 8, 6); *(b)* persistent or repeated noisy and/or broken data resulting from technical error (2, 3, 4, 8, 9, 10); *(c)* prolonged stationary data points reflecting stares (1, 6). Trials could be associated with more than one criterion.
